# Multivariate analysis and GIS approaches for modeling and mapping soil quality and land suitability in arid zones

**DOI:** 10.1016/j.heliyon.2024.e27577

**Published:** 2024-03-04

**Authors:** Mohamed E.M. Jalhoum, Mostafa A. Abdellatif, Elsayed Said Mohamed, Dmitry E. Kucher, Mohamed Shokr

**Affiliations:** a National Authority for Remote Sensing and Space Science (NARSS), Cairo, 11843, Egypt; b Department of Environmental Management, Institute of Environmental Engineering (RUDN University), 6 Miklukho-Maklaya St, Moscow, 117198, Russia; c Soil and Water Department, Faculty of Agriculture, Tanta University, Tanta, 31527, Egypt

**Keywords:** Multivariate analysis, Soil quality, Arid zones, GIS, Agricultural sustainability

## Abstract

Assessing soil quality marks the initial step in precision farming and agricultural management. Developing countries like Egypt face numerous hurdles in ensuring food security due to increasing populations and limited agricultural resources. A geographic information system (GIS) and multivariate analysis were utilized in the current work to evaluate and map a soil quality index (SQI). Moreover, the land suitability of the land for two plantations of the tree's oak (*Quercus robur*), and pine (*Pinus silvestris*), respectively was assessed using a parametric approach. A total of 82 soil profiles were selected to fulfill the objectives of the study. Based on the samples' PC scores, and agglomerative hierarchical clustering (AHC, the data was divided into two clusters: Cluster I and Cluster II, which collectively account for approximately 57% and 43% of the total data, respectively.. . The findings indicated that land suitability for planting *Q. robur* planted identified 2.14% of the research area as highly suitable (S1), 37.98% as moderately suitable (S2), and 59.89% as not suitable (N). Furthermore, the assessment of suitability for *P. silvestris* indicated that 50.88% of the investigated area was classified into: S1, 48.73% as S2, and 0.39% as N, which means it is not suitable for conservation activities. The research identified that soil depth beside excessive salinity and calcium carbonate as the primary soil constraints in the area in both clusters. The average soil depth, ECd and CaCO3 were 113.62 ± 12.41, 17.27 ± 10.23, 16.83 ± 6.57 in Cluster 1 and 45.43 ± 15.21, 22.42 ± 12.43, 21.55 ± 5.63 in Cluster II. The study demonstrates that integrating multivariate analysis with GIS enables a precise and streamlined assessment of the Soil Quality Index (SQI). Soil suitability modelling underscores the importance of implementing efficient management practices to attain agricultural sustainability in arid regions, particularly amidst intensive land utilization pressures

## 1 Introduction

The concept of soil quality (SQ) has become widely recognized and utilized for evaluating soil in diverse environmental systems [[1], [2], [3]]. Soil quality (SQ) is defined as soil's ability to provide plants with essential nutrients throughout their growth stages, crucial for maintaining agricultural productivity [4,5]. Hunger and poverty present significant global challenges for humanity, impacting over 800 million people globally. Africa carries the greatest burden of these challenges, holding the largest share globally [6]. Egypt's economy heavily relies on agriculture, which also provides the majority of its food security [7]. Drylands, encompassing various aridity levels such as dry subhumid, semi-arid, arid, and hyper-arid environments, are prevalent in regions where the aridity index falls below 0.65. These areas are inhabited by over 2 billion people, constituting 45.4% of the Earth's surface [8]. However, the harsh climate and limited land resources that dryland agroecosystems face have an adverse effect on the sustainability of crop production [9]. Hence, to ensure effective land use planning and the conservation of arable land resources, it is crucial to conduct thorough assessments and continuous monitoring of land attributes [10,11]. A major international objective nowadays is to maintain soils' overall quality [12]. This necessitates monitoring and evaluating soil-related characteristics, functions, and conditions in order to determine SQ. Additionally, it allows for the early identification of possible issues with degradation and the monitoring of changes as a result of practices related to land use and soil management [13]. The SQI typically indicates the regional variability of soil's chemical and physical characteristics l [14]. These physical, chemical, and biological characteristics of the SQI can be utilized to quantitatively investigate and quantify soil health and fertility status [15].

Physical indicators encompass factors such as soil depth, bulk density, porosity, aggregate stability, texture, and compaction. Meanwhile, chemical indicators include attributes like pH, salinity, organic matter content, phosphorus availability, cation exchange capacity, nutrient cycling, and the presence of soil contaminants [16]. While chemical indicators notify us of the existence of organisms, nutrient availability, water availability for plants, and movement of pollutants, physical indicators provide information on root growth, plant emergence speed, and water infiltration [15]. Therefore, by comprehending the soil quality, it is possible to delineate potential phases of successful soil management for sustainable agricultural production [17]. A variety of techniques, including both quantitative and qualitative approaches, have recently been developed for evaluating soil quality [2]. The diagnostic characteristics of soil are intricately tied to the comprehensive evaluation of soil quality, encompassing both quantitative and qualitative assessments. These attributes provide vital insights into the overall health and functionality of the soil, aiding in land management and environmental stewardship efforts [18]. Geographic information systems (GIS), a potent tool for measuring Soil quality (SQ), are among the contemporary monitoring strategies [19,20]. Specially referenced data can be edited, saved, and updated in the GIS. Furthermore, GIS can incorporate spatially referenced datasets for use in spatial modelling [21,22]. The application of GIS-geostatistical analysis empowers us to gain valuable insights into the spatial diversity inherent in soil data. Moreover, it enables the creation of seamless and detailed layers that are practically useful in the categorization and management of land resources. This approach is essential for informed decision-making, sustainable land use planning, and the efficient resources utilization [23,24]. The statistical analysis capabilities of GIS are crucial for forecasting at unsampled locations and for analyzing fluctuations in soil attributes [25]. Principal component analysis (PCA) and cluster analysis stand out as prominent multivariate analysis techniques extensively employed for the purpose of data identification, classification, and modelling. These analytical methods significantly contribute to enhancing our understanding of the data and are instrumental in guiding informed decision-making in various fields, including soil science and environmental management [26]. PCA is a statistical method that aids in reducing the number of attributes within a dataset by pinpointing the most crucial principal components (PCs) that encapsulate the majority of the data's information [27]. Where there are restrictions on data availability, a combination of statistical and geostatistical methods provides a practical and efficient solution [28]. The land's degree of suitability is determined by its capacity to yield crops and its resilience in the face of external conditions. The SQI measures the soil's capacity to support an ideal ecosystem that guarantees plant productivity, environmental quality, and sustainable human, animal, and plant health [29]. Egypt has 146 wastewater treatment facilities with a daily capacity of five million cubic meters [30,31]. Wastewater has been used to water trees along highways, city greenbelts, and woody production systems [32]. Egypt imports almost all of its wood, with minimal local supply from nut and fruit tree thinning, cutting, and urban plantings. Few natural tree species thrive in the largely desert region with little rainfall [33]. Human survival and development are based on agricultural productivity. Since there are less arable lands and a growing global population, sensible land use planning and management are necessary for effective production [34]. Land users could benefit from modelling cultivation priority planning as it would ensure appropriate land use, reduce existing difficulties, and support social, economic, and environmental goals like employment and sustainable land use [35] Thus, The main objective of the current work is to evaluate, characterize, and map the SQI in EastBeni-Suef in Egypt using an integrated multivariate analysis based on PCA and GIS. Moreover, assessing the suitability of land for oak (*Quercus Robur*) and pine (*Pinus Silvestris*) plantings based on physical, ecological, and climatic criteria. The outcomes of this investigation will assist in the long-term management of the region's land resources.

## 2 Materials and methods

### 2.1 Description of the study area

**Fig. 1:**
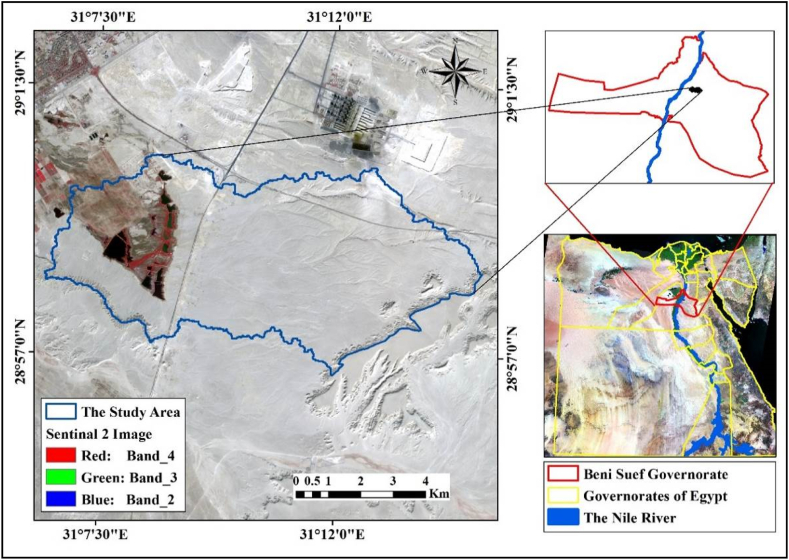
The location of the study area.

The study area is located in the eastern part of the Beni Suef Governorate within Egypt's Eastern Desert (see Fig. 1). Its boundaries are defined by longitudes 28° 56′ 40.2″E and 29° 00′ 21.9″E, and latitudes 31° 06′ 26.3″N and 31° 14′ 46.6″N.This area covers a total of 5500.9 ha (13592.6 acres). As a part of the Arabian-Nubian Shield, the area primarily consists of Precambrian basement rocks, which are predominantly of igneous and metamorphic origin. These foundational geological features are a fundamental characteristic of the region [36]. The geomorphology of the asea has rough topography that changes from limestone hills to relatively flat plains made of wind-blown sands to agricultural land near the Nile Valley [37]. Climate information from Beni Suef station reveals that the region experiences extremely hot summers and warm winters with cool evenings. According to the data, temperatures fluctuated between 19 °C and 36.7 °C from January to August, respectively,. In terms of humidity, August had the lowest mean at 36% and January had the highest average of 53%. Wind speeds ranged from 31 to 38 m/s [38].

### 2.2 Data collection

**Fig. 2:**
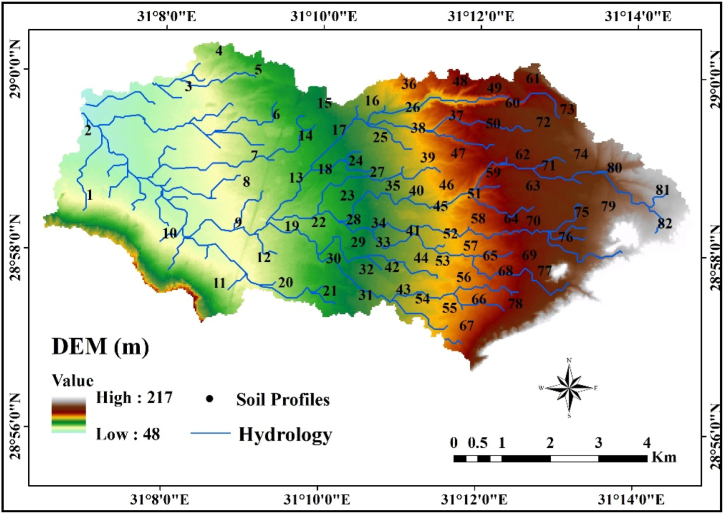
The soil sampling locations in the study area.

High-resolution multi-spectral optical imagery was produced by the Sentinal-2 data (S2A) satellite image from the European Space Agency (ESA) using 13 spectral bands of the MSI (multispectral imager) instrument with 4 bands at 10 m, 6 bands at 20 m, and 3 bands at 60 m spatial resolution [39]. SNAP software was used to resample all spectral bands at a resolution of 10 m [40] (S2A covering the study area were captured on March 29, 2022 (Fig. 1). Using ENVI image processing software the data were geometrically rectified and projected to the UTM zone 36 N coordinate system using the WGS 84 datum. The NASA—Climatology Resource for Agroclimatology website (http://power.larc.nasa.gov/) provided the climate data for the study area throughout the 36-year period (1985–2020). The source data for elevation from ESA was the Copernicus DEM (GLO-30 m) survey date January 01, 2011 to January 07, 2015 (Fig. 2). The DEM was then used to extract the necessary terrain characteristics, including relief intensity, slope, curvature, and elevation [41].

### 2.3 Map of the delineated landforms

ArcScene's was employed for the visual interpretation of European Sentinel 2 satellite images (S2A) dumped over the DEM created a 3D view for the extraction of the landform units. Errors and noise in the DEM were reduced by applying a straightforward filter based on focal neighborhood statistics. The reclassification of topographic parameters is what caused these noises. The central pixel within the moving window received the highest and average values from the neighboring pixels in the requested neighborhood. This operation was performed to calculate the majority and mean focal statistic values [42]. The method clearly showed the differences in altitude between each defined section. Utilizing this approach, we successfully distinguished between various landform units through visual interpretation of satellite imagery, 3D visualization of the Digital Elevation Model (DEM), and on-site field verification. This comprehensive process enabled us to accurately categorize and assign the appropriate nomenclature to these landforms.

### 2.4 Fieldwork and lab. Analysis

.Digging was carried out to a depth of 150 cm or until reaching the lithic contact in 82 geo-referenced soil profiles (Fig. 2), which were subsequently characterized [43]. Randomly selected soil profiles were used to depict the various landforms and landscape units in the study [44]. Disturbed samples were systematically collected from various horizons, carefully sealed within plastic bags to prevent any contamination or alteration, and subsequently transported to the laboratory for further analysis and examination. The disturbed samples were ground, air-dried, and sieved using a 2-mm mesh. The personnel conducting the soil survey stated that soil studies were carried out [45]. The particle size distribution using the pipette method, water retention capacity, and hydraulic conductivity were measured. Additionally The electrical conductivity (EC) and pH) of soil-water suspensions at a ratio of 1:2.5 were measured,. The calcium carbonate (CaCO_3_) was calculated using the calcimeter method.

### 2.5 Statistical analysis

The minimum, maximum, arithmetic mean, and standard deviation of the examined soil parameters were computed using SPSS version 25. Furthermore, the linear relationships between these variables were explored using the Pearson correlation coefficient (r). Kaiser–Meyer–Olkin (KMO) was used to assess the sampling adequacy for the entire data set because this analysis requires adequate sampling. The PCA would be appropriate if the KMO value was higher than 0.50. Furthermore, the Bartlett's test was run; if the test's p value is less than 0.05, the PCA might be appropriate for this work. The PCA used calculated using SPSS version 25. PCA was utilized to transform the dataset into different variables referred to as principal components (PCs), serving the dual purpose of simplifying the dataset and averting issues related to multicollinearity among the original variables. A large portion of the variation shown in the original variables can be explained by these PCs. PCs with eigenvalues greater than 1.0 were the only ones taken into [46]. Based on the PCA results, the soil samples that were taken into consideration as assessment objects for soil quality were categorized using the AHC methods into different clusters, with each location being defined by a set of soil factors (chemical, physical, and biological). This approach led to the graphic arrangement of dissimilar groupings of soil variables in a structure known as a dendrogram of dissimilarity [47].

### 2.6 Calculation of soil quality index (SQI)

Soil Quality Index (SQI) Calculation and Mapping [48].

(1) $$SQI=\sum_{i=1}^{n}Wi\times si$$

where Wi is an indicator's weight and Si is an indicator's score (Table S1), n is the total number of indicators. According to the commonality of each indicator determined by employing mathematical statistics through factor analysis in IBM SPSS Statistics 25, weight values were assigned to each parameter. Each parameter's weight value was calculated as a ratio of its commonality to the total commonality [49].

### 2.7 Soil quality grads

Each indicator was given a grade, with the highest being very good (Grade I), followed by good (Grade II), fair (Grade III), and bad (Grade IV). Each interval's width was established by dividing the range of values for each index into four equal parts. Adding this amount to each index's lowest value [50].

### 2.8 Spatial variability of soil characteristics

Inverse distance weighting (IDW), is a type of deterministic interpolation technique. The IDW approach can be characterized as a distance reverse function of each point from neighboring points, and it assumes that the rate of similarities and correlations between neighbors is proportionate to their distance from one another [51]. Equation (2) illustrates how the inverse distance weighted (IDW) tool in ArcGIS 10.4 was used to construct the interpolation of soil properties. The locations of soil profiles were digitally recorded to determine the weighted average value for each parameter that is specific to each soil profile (soil depth, WHC, HC, pH, EC, CaCO_3_, sand, silt, and clay%). The Inverse Distance Weighting (IDW) method is commonly applied in soil research due to its simplicity and user-friendliness [[52], [53], [54], [55]]. Distance lessens the local impact of the measurement location, as shown by the following equation [56].

(2) $$Z_{p}=\left(\sum_{i=1}^{n}\left(\frac{zi}{di}\right)/\sum_{i=1}^{n}\left(1/di\right)\right)$$

where zp is the value predicted at point P, zi is the z value at the measured point i, and di is the distance between point 0 and the point ‘i’.

Since these data were not normally distributed, the majority of the interpolation maps were created using the geometrical interval classification approach [57].

### 2.9 Assessment of land suitability index (LSI)

The land suitability index is computed according to Ref. [58]. The geometrical mean value of the scores provided to each land quality or characteristic and climatic rate in the interaction of the nth root values of scores is multiplied to determine the land index of each land unit using the formula below [58]:

(3) $$LSI={\prod_{i=1}^{n}Xi}^{\left(\frac{1}{n}\right)}*\sqrt{\frac{\prod_{i=1}^{n}Xi}{100^{n}}}$$

where *LSI* is the land suitability index, X is the score assigned to each land characteristic, and n is the number of land attributes or features. Potential land suitability for oak and pine in the study area was determined by comparing Egyptian conditions zones with the crop requirements [58] (Table S2).

## 3 Results and discussion

### 3.1 Surface parameter

**Fig. 3:**
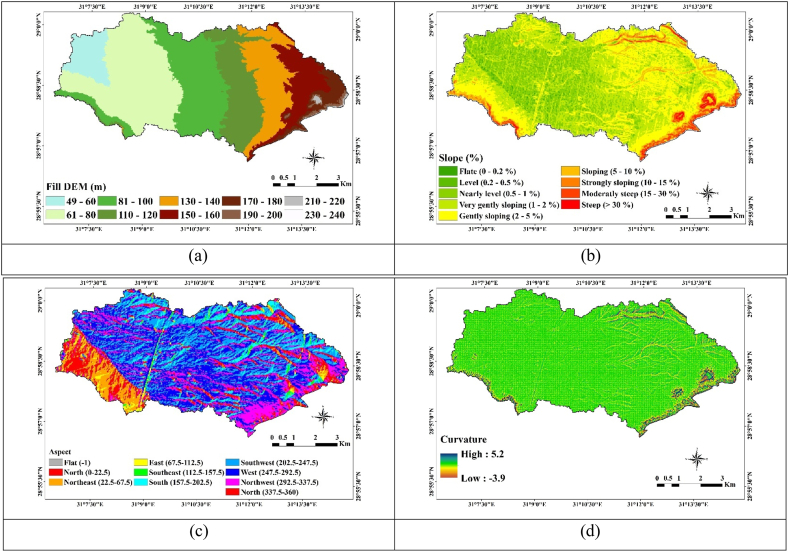
Some topographic parameters (a) Filled-dem, (b) slope, (c) aspect, (d) curvature.

The assessment of soil qualities incorporated considerations for both topography and land use. This is essential because topography and land use exert a significant influence on soil characteristics [59]. The surface parameter was utilized to create the suitability model and landform units, (Fig. 3a–d). The fill function technique was employed to smooth irregular values by assigning average values from neighboring cells (Fig. 3a). Elevation increases gradually from west to east, reaching a maximum height of 217 m above sea level (m.a.s.l) and the minimum elevation is 46 m.a.s.l.

Slope and aspect were extracted from the digital elevation model (Fig. 3b and c). The slope ranges from flat to steep and the derived aspect ranges in eight directions, rotating from 0 to 360, were also recognized, in addition to the flat area. Aspect can vary the intensity of solar radiation and create microclimates to have an impact on soil organic matter. This, in turn affects soil aggregate stability and soil erodibility factor [60,61]. Conversely, regions where deposition occurs, typically on concave slopes or gentle to moderate slopes, have deeper topsoil depth, greater vegetation and organic matter content, stable soil structure, enhanced permeability and infiltration capacity, and lower erodibility. As a result, concave slopes experience less erosion [62], whereas convex slopes are more easily deteriorated and highly erodible [63]. Curvature is a degree of surface roundness, varied between −3.9 at the lowest point and 5.2 at the highest one (Fig. 3d). A negative value in the wade reflects the surface's upward convex shape.

### 3.2 Geomorphological of the study area

**Fig. 4:**
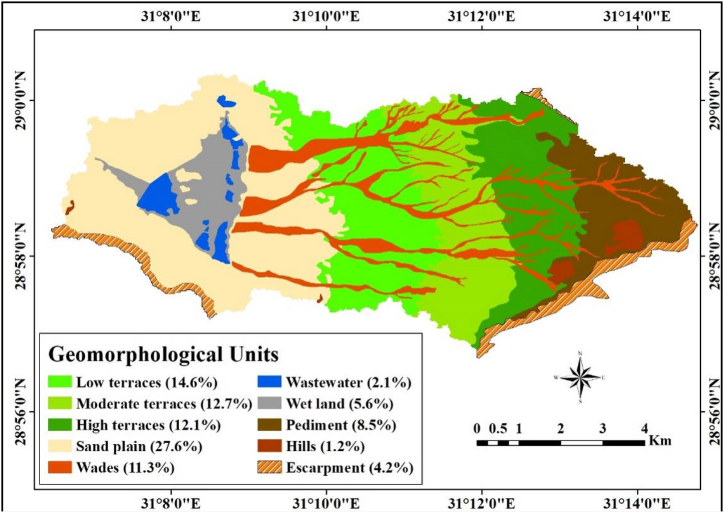
Geomorphologic units for the study area and the percentage of their areas.

Geomorphology is the study of landforms, focusing on their nature, origin, development, and material composition [64]. It examines the landforms that sculpt the surfaces of the earth, emphasizing their creation and the context of their surroundings. This is e explored by highlighting their formation and development processes, as well as their relationship to their surroundings [65]. Landform units were derived from DEM and S2A, and Fig. 4 depicts the major ten landforms. Sandplains occupy the western part of the study area. This landscape covers about 1518.1 ha (27.6%) of the area. Along the Nile Valley, desert sands have accumulated in the soils [33]. The Nile Valley gradually widens northward, from a few hundred meters in the south to 23 km at the latitude of Beni Suef city, located about 122 km south of Cairo) [66].

Terraces are present in the eastern portion of the study area, further categorized into high, moderate, and low terraces, collectively covering a significant portion of the total area, approximately 2163.7 ha, which constitutes 39.4% of the land. These landforms cover an area of 664.5, 698.3, and 800.9 ha of study area respectively. Terraces are formed through the cyclic process of erosion and the deposition of alluvial sediments (cut and fill) in an environment that gives rise to a staircase-like formation [67]. Wades, pediment, and wetland units cover an area of 624.3 ha (11.3%), 469.7 ha (8.5%), and 308.7 ha (5.6%) of the study area, respectively. Other notable features include escarpments, hills, and wastewater with an area of about 146.4 ha (7.5%).

### 3.3 Spatial distribution of soil properties

**Fig. 5:**
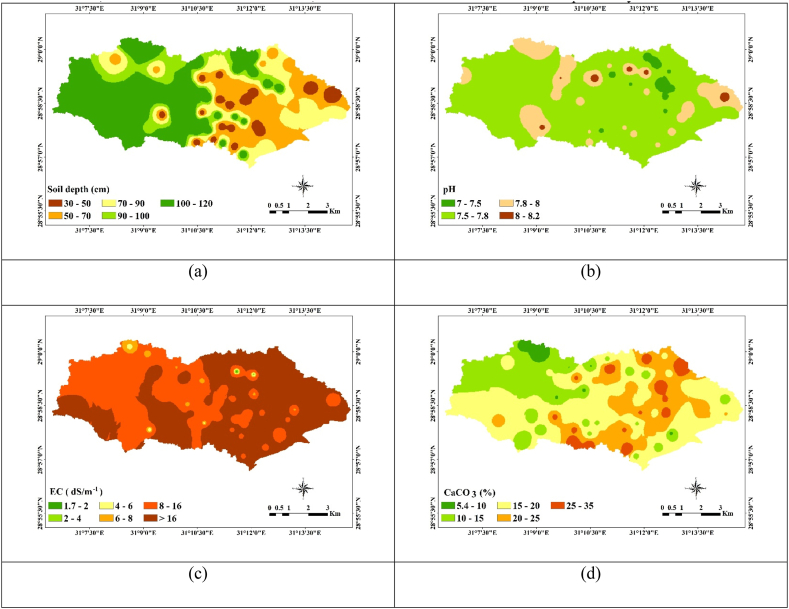

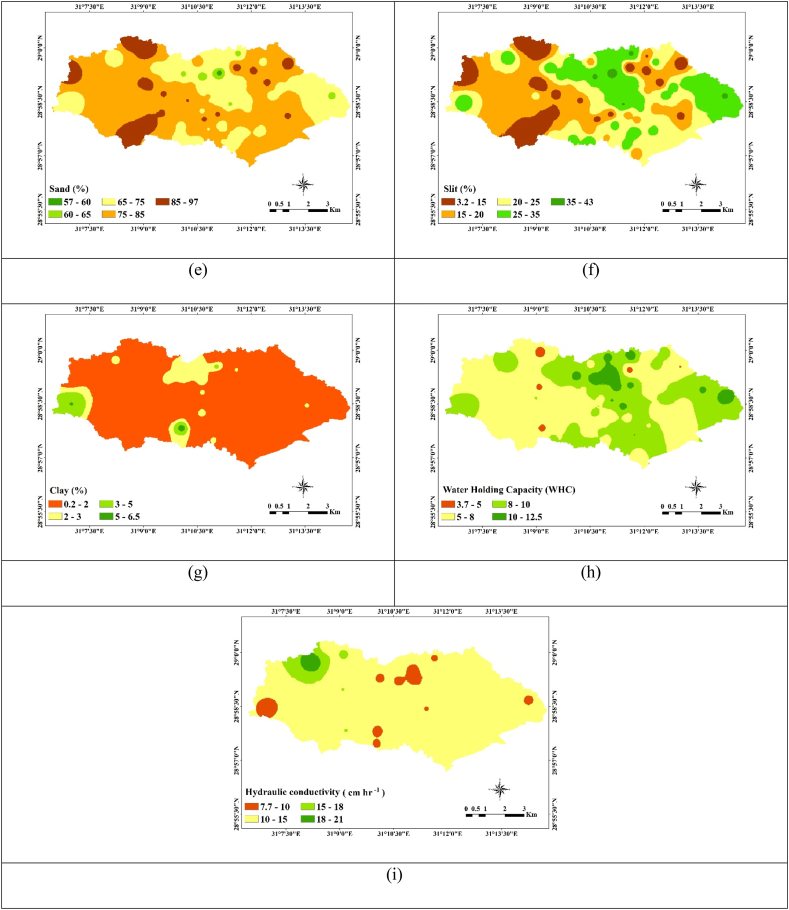
Interpolation maps of some soil properties, (a) soil depth (cm), (b) pH, (c) EC ds m^−1^, (d) CaCO_3_ %, (e) sand% (f) silt, (g) clay %, (h) WHC %, and (i) HC.

The main soil group in the research region, as determined by the WRB, is ARENOSOLS [68]. The qualifier “Arenic,” which denotes a loamy sand or finer texture over the top 50 cm of soil.The following soil units have been found in the research area: carbonates (Calcisols), soils with accumulated soluble salts (Solonchaks) and having non developed diagnostic horizons (Regosols) Table 1 provides statistical analyses and interpolated maps of mean-weighted soil properties. The visual representation of this data is depicted in (Fig. 5a–i). The soil depth was very shallow to deep, it ranged between (30–120 cm) (Fig. 5a). pH values ranging between 7.3 and 8.2, indicating that,most study areas are slightly alkaline (Fig. 5b). The increase in pH results in the soil's organic and inorganic colloids becoming more negatively charged, thereby improving the soil's ability to hold metal cations through electrostatic sorption [69]. In this region, the electrical conductivity (EC) values vary from 1.6 to 51 dS m^−1^, indicating a spectrum of soil salinity levels, ranging from slightly saline to very strongly saline soils (Fig. 5c). Salinized soils are predominantly located in drylands because of the arid climate and high rates of evaporation [70].Consequently, addressing high-salinity soils calls for the implementation of a recommended management strategy. One effective approach involves leaching excess salts from the soil through the utilization of high-quality irrigation water, which aids in improving soil quality and fertility [71,72].

**Table 1:** Statistics of some mean weighted soil properties.

Soil variables	EC (dS m^−1^)	pH	CaCO_3_	Sand	Slit	Clay	WHC	H. C (cm h^−1^)
			(%)					
Min.	1.63	7.31	5.40	57.3	3.30	0.20	3.70	7.70
Max.	51.25	8.17	34.80	96.50	42.30	6.40	12.50	21.10
Average	19.50	7.70	18.90	76.80	22.00	1.16	8.10	12.10
STD	11.50	0.17	6.50	8.90	8.70	1.10	1.90	2.10

Note Min., Max. and STD are Minimum, Maximum and stander deviation respectively.

The CaCO_3_% values range from 5.4% to 34.8% (Fig. 5d); Elevated concentrations of CaCO_3_ are linked to the existence of parent materials abundant in shell fragments, which appear in the east area. In certain regions, the significant CaCO_3_ content can lead to the formation of extremely compacted layers that impede both water infiltration and the growth of crop roots. Additionally, calcareous soils with elevated CaCO_3_ levels may exhibit phosphate fixation, affecting the availability of phosphorus in fertilizers. This underscores the need for soil management practices to address these challenges and ensure optimal crop growth. The authors observed that calcareous soils had good P retention, even if organic matter (OM) application made it more mobile [73]. The soil texture differs between sandy and sandy loam (Fig. 5e–g). The WHC% content of the study area ranges between 3.7 and 12.5 % and averages 8.1% (Fig. 5h). The coarse texture causes a relatively low water retention capacity [24]. Hydraulic conductivity (H.C) is a property of the soil related to its ability to transmit water and depends on the soil type, porosity, and permeability [74]. The higher value of hydraulic conductivity for soil, the more permeable it is to water. The H.C % content in the study area varies between 7.7% and 21 with an, average is 12.1 % for most study areas (Fig. 5i). The variance of sand%, as measured by the standard deviation of the soil characteristics, is quite large (STD = 8.90), followed by silt% (STD = 8.70), and CaCO_3_ (STD = 6.5). Conversely, there is moderate variance in clay%, WHC, and HC (STD = 1.1, 1.9, and 2.1, respectively) [46].

*3.4.PCA* of studied variables Table 2 provides an overview of PCA's findings. Considering that the eigenvalues of the first three Principal Components (PCs) are greater than 1, Kaiser's approach [75] was employed to select these PCs, excluding the remaining ones. As a result of these findings, 71.97% of the total variation can be explained by the first three PCs. The first PC, which explains 37.90% of the total variance, shows stronger positive correlations with EC, CaCO_3_, and WHC, while the second PC, which explains 19.32% of the total variance, is closely associated with pH and HC. Depth, clay and slope are associated with the third PC, representing 14.47% of the overall variation.

**Table 2:** The factors that PCA extracted.

Properties	PCs		
	1	2	3
Depth (cm)	−0.500	0.266	**0.584**
pH	−0.552	**−0.584**	0.155
EC (dS cm^−1^)	**0.798**	0.417	0.016
Clay %	0.255	−0.450	**0.740**
CaCO_3_ %	**0.798**	0.234	0.091
WHC %	**0.736**	−0.500	−0.137
HC cm h^−1^	−0.713	**0.505**	−0.084
Slope %	0.299	0.444	**0.483**
	Eigenvalue	Variance (%)	Cumulative (%)
	3.03	37.90	37.90
	1.54	19.32	57.22
	1.18	14.74	71.97

Values in bold correspond for each variable to the factor for which the squared cosine is the largest.

The findings of the KMO test of sample adequacy and Bartlett's test of sphericity are displayed in Table S3. In accordance with [76], the KMO value is larger than 0.6 and the significance level of Bartlett's test of sphericity was 0.0001, indicating that the sample size is enough for determining the factor structure. These tests' findings indicate that the variables are not entirely uncorrelated; the model's variables can describe the phenomenon, making Principal Component Analysis appropriate. The research revealed a strong inverse relationship between soil pH and EC (r = −0.64) in the same line [77] (Table 3). Additionally, a statistically significant negative association (*P* < 0.05) exists between soil depth and EC, likely due to variations in groundwater depth, which substantially impacts soil salinity and increases as the water table decreases [78]. According to numerous researchers [[79], [80], [81]], higher EC often indicates higher clay percentage and lower sand content (Table 3). The correlations between soil texture and EC range from weak to very strong (coefficient of determination approaching 1), with a correlations coefficient of r = 0.07 Soil moisture and electrical conductivity also exhibit a high correlation [82], with a correlation coefficient of 0.390 between EC and WHC Overall, the interaction between different land use types, slope, aspect, and soil physicochemical qualities had a substantial impact [83].The slope exhibits a statistically significant positive connection with EC and CaCO_3_ (r = 0.39, 0.21 respectively). Clay has been shown to have a positive and substantial correlation with WHC (r = 0.23). However, it has also shown that WHC and pH have a negative association (r = -0.19) as noted in Ref. [84]. Coarser-grained soils have higher hydraulic conductivity (HC) at high volumetric moisture content levels, whereas finer-grained soils have lower hydraulic conductivity [85]. Consequently, there is a statistically significant inverse association between the HC and the clay % (r = −0.34) (Table 3).

**Table 3:** The analyzed variables' Pearson correlation matrices.

Variables	Depth	pH	EC	Clay	CaCO_3_	WHC	HC	slope
Depth	1	0.039	−0.238∗	0.102	−0.367**	−0.467**	0.290	0.043
PH	0.039	1	−0.641**	0.157	−0.411**	−0.196	0.156	−0.160
EC	−0.238∗	−0.641**	1	0.076	0.692**	0.340**	−0.337**	0.291**
Clay	0.102	0.157	0.076	1	0.199	0.234∗	−0.342**	0.069
CaCO_3_	−0.367**	−0.411**	0.692**	0.199	1	0.346**	−0.361**	0.318**
WHC	−0.467**	−0.196	0.340**	0.234∗	0.346**	1	−0.762**	0.010
HC	0.290**	0.156	−0.337**	−0.342**	−0.361**	−0.762**	1	−0.045
Slope	0.043	−0.160	0.291**	0.069	0.318**	0.010	−0.045	1

∗ P = 0.05, **. P = 0.01.

### 3.4 Cluster analysis

**Fig. 6:**
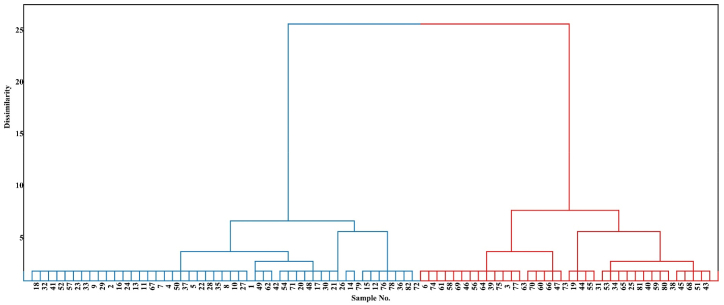
Dendrogram for the recovered clusters by agglomerative hierarchical clustering (AHC).

**Fig. 7:**
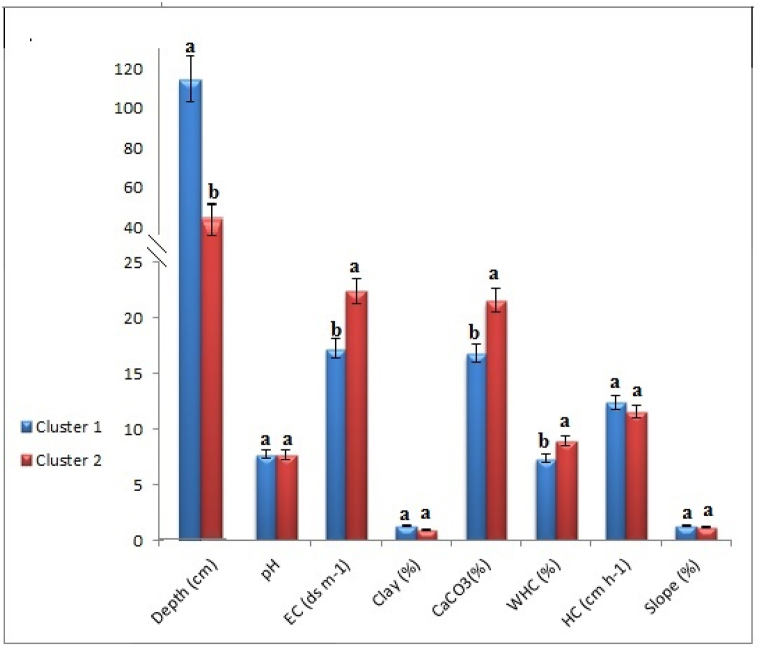
Shows the significance levels of the variables under study. The lettered discrepancies between the two variables indicate a significant difference in their means.

Cluster analysis is a valuable technique for resolving classification issues, aiming to arrange variables so that there is a strong degree of linkage between members of the same cluster and a weak degree of correlation between members of other clusters [86]. The division of several variables into discrete groups using clustering, a productive statistical technique for handling data [25]. Each group represents a certain kind of soil quality. The PC scores of the samples were used to identify two data clusters (Fig. 6). Cluster I comprises around 57% of the overall data, whereas Cluster II accounts for 43%. PCA is employed to categorize the variables under examination into distinct groups, whereas cluster analysis is utilized to assemble data into clusters. In cluster analysis, the data points within a cluster exhibit higher similarity to each other compared to those in other clusters [87,88]. In this study, agglomerative hierarchical clustering (AHC) was applied to partition the data into two distinct groups or clusters. The dendrogram presented in Figure showcases the extent of dissimilarity between these two clusters, each possessing unique characteristics. The statistical summary provided in Table 4 reveals that there are 47 observations in the first cluster and 35 in the second. For every variable, there are variations in the means, ranges, and standard deviations (STD) for every cluster. Those two groups were extracted using the criteria. The gathered data showed that the two clusters differed significantly in terms of depth, EC, CaCO_3_, and WHC Fig. 7. Between the two clusters, there were no discernible differences in pH, HC, or slope. Therefore, cluster I can be identified from cluster II by its greater depth, lower EC, lower CaCO_3_, and higher SQI.

**Table 4:** Quantitative descriptive statistics of the investigated variables for clusters.

Cluster 1						Cluster 2				
Variables	NO of observation	Min.	Max.	Mean	STD	NO of observation	Min.	Max.	Mean	STD
Depth (cm)	47	90	120	113.62	12.41	35	30	60	45.43	15.21
pH		7.31	8.10	7.72	0.167		7.32	8.17	7.68	0.18
EC (ds m^−1)^		1.63	41.9	17.27	10.23		6.02	51.2	22.42	12.83
Clay (%)		0.20	6.44	1.30	1.23		0.33	2.68	0.96	0.63
CaCO_3_(%)		5.39	33.28	16.83	6.57		9.02	34.83	21.55	5.63
WHC (%)		3.73	11.33	7.35	1.90		4.95	12.50	8.97	1.68
HC (cm h^−1)^		7.71	15.47	12.39	1.87		8.61	21.13	11.53	2.14
Slope (%)		0.15	6.80	1.30	1.19		0.29	2.77	1.18	0.47
SQI		Fair					bad

### 3.5 Soil quality index mapping

**Fig. 8:**
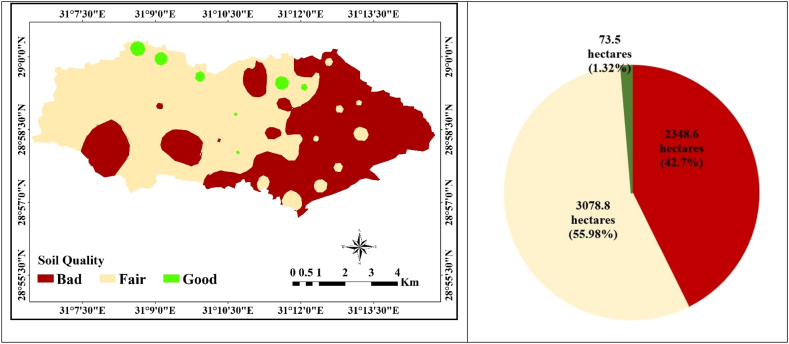
Interpolation map of SQI within study area.

To map the spatial distribution of soil quality in the study area, we leveraged the SQI results obtained by Equation (1) through the use of IDW interpolation. This interpolation technique allowed us to generate a comprehensive representation of the variability in soil quality across the region. A table displays the findings, where the SQI yields scores between 0.38 and 0.54. As seen in Fig. 8 and Table 5, the SQI is divided into three quality classes. The soil's composition as well as the local climatic and environmental conditions have an impact on it [89,90]. The first class is distinguished by a poor quality index and accounts for around 42.7% (or 2348.6 ha) of the total area. The second class, which comprises around 55.98% of the study area (3078.8ha), is distinguished by fair soil quality. Most of the study area was categorized as having fair soil quality, with the average values of soil variables in this category, including depth, pH, EC, clay content, and CaCO_3_, WHC, and slope were 96.43 ± 33.02, 7.74 ± 0.16, 14.67 ± 7.65, 1.10 ± 1.02, 16.82 ± 4.24, 8.04 ± 1.87, 11.99 ± 1.68 and 1.01 ± 0.66 respectively. The third class, which spans roughly 1.32% (173.5 ha), is good soil quality.The SQI of the research area is negatively impacted by low clay content values and high EC values [6]. Micro-irrigation with drip and sprinkler systems is advised for all classess. Micro-irrigation increases the yield of crops and the efficiency with which water and fertilisers are used as compared to surface approaches [91,92]. Additionally, it makes it possible to manage issues with lime, salinity, sodicity, coarse texture, and sloping surfaces. Physical indicators such as depth, bulk density, porosity, aggregate stability, texture, and compaction exert an influence on the arrangement and structure of soil particles. and pores, which explains how they affect root growth, plant emergence speed, and water infiltration [93]. The shift in quality classes aligns with a decline in limit values from the eastern to the western regions of the research area, as evidenced by the spatial distribution of these classes. This pattern indicates a gradient in soil quality across the region, with the eastern part featuring higher-quality soils and the western part exhibiting lower-quality soils. This variability holds considerable significance for land use and agricultural planning in the area. (Fig. 8). Firdous et al., 2016 [94] demonstrated tha the value of multivariate statistical methods for the examination and comprehension of intricate datasets to Recognise differences in soil quality.

**Table 5:** Averages of the investigated soil variables for each SQI of the research region.

SQI	Average values								Area	
Classes	Depth (cm)	pH	EC (ds m-1)	Clay (%)	CaCO_3_(%)	WHC (%)	HC (cm h-1)	Slope (%)	ha	%
Bad	70.67 ± 35.70	7.66 ± 0.16	24.84 ± 11.70	1.13 ± 1.08	21.71 ± 6.49	8.39 ± 1.90	11.79 ± 2.25	1.44 ± 1.13	2348.6	42.7
Fair	96.43 ± 33.02	7.74 ± 0.16	14.67 ± 7.65	1.10 ± 1.02	16.82 ± 4.24	8.04 ± 1.87	11.99 ± 1.68	1.01 ± 0.66	3078.8	55.98
Good	116.25 ± 10.60	7.79 ± 0.15	8.32 ± 3.36	1.37 ± 0.87	10.80 + 3.39	6.62 + 1.79	13.19 + 1.53	0.92 + 0.33	73.5	1.32

### 3.6 Land suitability (LS) zonation

**Fig. 9:**
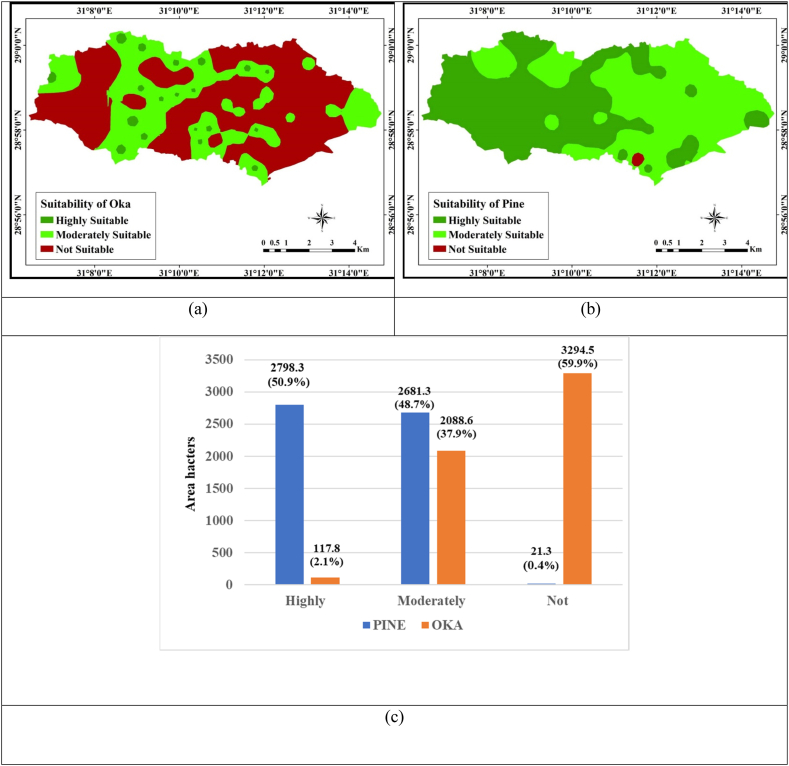
Interpolation map of (a) oak (*Quercus Robur*), (b) pine (*Pinus Silvestris*) trees suitability in the study area, (b) areas of land suitability for trees.

A land suitability evaluation can help determine the optimum way to manage of land so that, agricultural yield is maximized while maintaining the ability of natural resources, such soil, to support growth, is not compromised [95]. Using a parametric approach, the land suitability index values for Oka (*Q. robur*)and (Pine) *P. silvestris* plantings ranged from 19 to 84 and 20 to 84, respectively (Fig. 9a and b). For both tree species, the land compatibility in the research region's were categorized as Highly Suitable, Moderately Suitable, and Not Suitable. Approximately 2.14 percent (117.8 ha) of the study area was classified as very suitable, 37.98 percent (2088.6 ha) as moderately suitable (S2), and 59.89 percent (3294.5 ha) as not suitable (N) of the study area, according the zonation of land suitability for Q. robur cultivation (Fig. 9c). The zonation map for P. silvestris plantation's indicated that 50.88% of the research area (2798.3 ha) was classified as S1, 48.73% of the area (2681.3 ha) as S2, and 0.39% of the area (21.3 ha) as not suitable for conservation practices (N) (Fig. 9c). Excessive salinity and calcium carbonate were identified as the main soil limiting variables. Wherever possible, mitigating the impact of these elements improved the land suitability for all selected trees. Among the various soil management strategies recommended to mitigate soil salinity, the application of low-salinity water to facilitate salt leaching from the soil root zone stands out as a promising technique. This approach involves the controlled use of water with reduced salinity levels to help flush salts from the root zone of the soil, thereby improving its quality and reducing salinity-related issues. It's a practical method employed in addressing salinity challenges in agricultural and land management [71]. Plant species exhibit significant differences in the rate of growth when exposed to salt stress [96].

## 4 Conclusion

To categorize SQI within the study area, integrated PCA and AHC analysis was utilized, depending on associations and interactions between soil variables. Furthermore, the current study used parametric methodologies to assess the suitability of the investigated area for oak and pine forest plantations agricultural practices and climatic factors have a significant impact on the physical, chemical, and fertility characteristics of soil, ultimately affecting soil quality (SQ). It is crucial to comprehend the ways in which agricultural methods and climate conditions influence these fundamental soil attributes to facilitate effective land management and promote sustainable agricultural practices. According to the PCA results, the first PC, which accounts for 37.90% of the total variance, has stronger positive correlations with the variables EC, CaCO_3_, and WHC, whereas the second PC, which accounts for 19.32% of the total variance, is significantly correlated with the variables pH and HC. The third PC, which accounts for 14.47% of the overall variation, is related to depth, clay, and slope. Two clusters were created from the soil data: Cluster I accounts for nearly 57% of the total data, and Cluster II for 43%. For both tree species, the compatibility of the land was rated as Highly, Moderately, and Not Suitable. 2.14% (117.8 ha) of the research area was zoned as highly suitable, 37.98% (2088.6 ha) as moderately suitable (S2), and 59.89% (3294.5 ha) as not suitable (N) for a Q. robur plantation, according the zonation of land suitability for Q. robur plantation (Tables and). According to the zonation map for the P. silvestris plantation, 50.88% of the research area (2798.3 ha) was classified as S1, 48.73% of the area (2681.3 ha) as S2, and 0.39% of the area (21.3 ha) as N, which is not suitable for conservation techniques. The differences and spatial disparities in Soil Quality Index (SQI) and Land Suitability (LS) across different locations were promptly revealed through the application of GIS for soil parameter mapping. In general, the results underscore that the integration of PCA and GIS offers a precise and effective method for assessing both SQI and LS. In order to close the gap between production and consumption, it is crucial to regularly evaluate the quality of the soil, identify the factors that limit SQ, and maintain high crop yields. Future research should consider expanding fieldwork and improving methods for calculating soil quality and land suitability. Funding: “This research received no external funding

## Data availability statement

All data are included in the manuscript and supplementary files.

## CRediT authorship contribution statement

**Mohamed E.M. Jalhoum:** Software, Formal analysis, Data curation, Conceptualization. **Mostafa A. Abdellatif:** Writing – review & editing. **Elsayed Said Mohamed:** Writing – review & editing, Supervision. **Dmitry E. Kucher:** Writing – review & editing, Funding acquisition. **Mohamed Shokr:** Writing – review & editing, Writing – original draft, Validation, Software, Methodology, Investigation, Formal analysis, Data curation, Conceptualization.

## Declaration of competing interest

The authors declare that they have no known competing financial interests or personal relationships that could have appeared to influence the work reported in this paper.
