# Supplementary material for: Multivariate analysis and GIS approaches for modeling and mapping soil quality and land suitability in arid zones
Source: Heliyon. 2024 Mar 4;10(5):e27577. doi: 10.1016/j.heliyon.2024.e27577 (PMC10923861; doi:10.1016/j.heliyon.2024.e27577)
Supplement: Multimedia component 1 [file mmc1.docx]

Table S1 Scores of all variables

| **Indicator** | **Description** | **Threshold** | **Score** | **Weight** |
| --- | --- | --- | --- | --- |
| pH | Neutral | 6.6-7.3 | 1.0 | 0.11 |
|  | Slightly alkaline | 7.3-7.8 | 0.8 |  |
|  | Moderately alkaline | 7.8-8.4 | 0.6 |  |
|  | Strongly alkaline | 8.4-9 | 0.4 |  |
|  | Very strongly alkaline | >9 | 0.2 |  |
| EC (dS m^-1^) | Non-saline | <2 | 1.0 | 0.14 |
|  | Very slightly saline | 2-4 | 0.8 |  |
|  | Slightly saline | 4-8 | 0.6 |  |
|  | Moderately saline | 8-16 | 0.4 |  |
|  | Strongly saline | >16 | 0.2 |  |
| CaCO_3_ (%) | Non-calcareous | 0 | 0.2 | 0.12 |
|  | Slightly calcareous | 0-2 | 1.0 |  |
|  | Moderately calcareous | 2-10 | 0.8 |  |
|  | Strongly calcareous | 10-25 | 0.6 |  |
|  | Extremely calcareous | >25 | 0.4 |  |
| Depth | Very deep | >150 | 1.0 | 0.11 |
|  | Deep | 150-100 | 0.8 |  |
|  | Moderately deep | 100-50 | 0.6 |  |
|  | Shallow | 50-30 | 0.4 |  |
|  | Very shallow | <30 | 0.2 |  |
| Texture | Clay loam, |  | 1.0 | 0.14 |
|  | Silty clay loam, loam, silty clay, silt |  | 0.9 |  |
|  | Silt loam, clay < 60% |  | 0.8 |  |
|  | Sandy clay, sandy clay loam, sandy loam |  | 0.7 |  |
|  | Clay > 60% |  | 0.6 |  |
|  | Loamy sand |  | 0.4 |  |
|  | Sand |  | 0.2 |  |
| Water holding capacity (WHC) % | Very High | >35 | 1.0 | 0.03 |
|  | High | 35-30 | 0.8 |  |
|  | Moderate | 30-20 | 0.6 |  |
|  | Low | 20-15 | 0.4 |  |
|  | Very low | <15 | 0.2 |  |
| Hydraulic conductivity (HC), cm hr^-1^ | Low | 1-2 | 1.0 | 0.13 |
|  | moderate | 2-6 | 0.8 |  |
|  | Very low | 0.05-1 | 0.6 |  |
|  | High | 6-12 | 0.4 |  |
|  | Extremely low- very high | <0.05 or >12 | 0.2 |  |
| Slope, % | Very gently sloping | <2 | 1.0 | 0.09 |
|  | Gently sloping | 2-5 | 0.8 |  |
|  | Sloping | 5-10 | 0.6 |  |
|  | Strongly sloping | 10-15 | 0.5 |  |
|  | Moderately steep | 15-30 | 0.4 |  |
|  | steep | >30 | 0.2 |  |

**Sources:** FAO 2006, Soil Science Division Staff, 2017, Yao et al.,2013 and Hazelton and Murphy, 2016

**Table S2** Climate, terrain, and soil requirements for Oak and Pine plantation.

| **Criteria** | **Index** | **Degree of limitation (Oka)** | | | **Degree of limitation (Pine)** | | |
| --- | --- | --- | --- | --- | --- | --- | --- |
|  |  | **S1 (100-75)** | **S2 (75-50)** | **N (50 -0)** | **S1 (100-75)** | **S2 (75-50)** | **N (50 -0)** |
| **Climate** | Minimum Temp. | > (-20) | (-20) - (-25) | < (-25) | (-35) | (-35) - (-45) | < (-45) |
|  | Maximum Temp. | 27-15 | 14-15 | <14 | 25-14 | 14-12 | <12 |
|  | Annual precipitation | 750-500 | 500-250 | < 250 | >700 | 700-500 | <500 |
| **Land Terrain** | Elevation (m) | 600 - 800 | 800-1200 | > 1200 | <600 | 600 - 700 | > 700 |
|  | Slope (%) | 0- 10 | 10-30 | >32 | 10 - 56 | 3 - 10 | < 3 |
|  | Aspect | N, NW, NE, Flat | W, E | S, SE, SW | N, NW, NE, Flat | W, E | S, SE, SW |
| **Soil characteristics** | Soil texture | SiCL, CL, Si, SiL, SC, L, SCL | SiC, SL, LS | S, C | SiCL, CL, Si, SiL, SC, L, SCL, SL | SiC, LS, S | C |
|  | Coarse fragments (%) | 0 - 15 | 15 - 60 | 60-100 | 0 - 40 | 40 - 80 | > 80 |
|  | Soil depth (cm) | 200 - 100 | 100 -50 | 50- 0 | > 100 | 100 - 30 | 30-0 |
|  | Drainage | W, MW, I | SE, P | E, VP | W, MW, I | E, SE, P | VP |
|  | CaCO_3_ | 0 - 10 | 10 -30 | >30 | 0 - 30 | > 30 | .. |
|  | ECe (dS/m) | 0 -2 | 2 - 4 | > 4 | 0 - 8 | 8 - 10 | >10 |
|  | pH | 7.5 - 5.5 | 5.5 - 4.5 | < 4.5 | 6.5 - 5.5 | 5.5 - 4.0 | < 4.0 |
|  |  |  | 8.5 - 7.5 | > 8.5 |  | 8.5 - 6.5 | > 8.5 |

**Source**: Amin et al., 2020

Table S3. The Kaiser–Meyer–Olkin (KMO) and Bartlett sphericity tests

| **KMO and Bartlett's Test** | | |
| --- | --- | --- |
| Kaiser-Meyer-Olkin Measure of Sampling Adequacy. | | 0.642 |
| Bartlett's Test of Sphericity | Approx. Chi-Square | 240.186 |
|  | df | 28 |
|  | Sig. | 0.0001 |

References

FAO. Guidelines for Soil Description, 4th ed.; Food and Agriculture Organization of the United Nations (FAO): Rome, Italy, 2006.

Amin Gholizadeh, A.; Bagherzadeh, A.; Keshavarzi, A. Model application in evaluating land suitability for OAK and PINE forest plantations in Northeast of Iran. J. Geology, Ecology, and Landscapes, 2020, 4 (3), 236–250. https://doi.org/10.1080/24749508.2019.1633217.

1-Soil Science Division Staff,. Soil survey manual. USDA Handbook 18. Government Printing Office, Washington, D.C, USA, 2017

2-USEPA (United States Environmental Protection Agency), 1995. Test methods for evaluating solid waste. Vol. IA: Laboratory manual physical/chemical methods, SW 846,3rd ed. U.S. Gov. 3-Print. Office, Washington, DC. Yao, R.-J., Yang, J.-S., Zhang, T.-J., Gao, P., Yu, S.-P., Wang, X.-P.,. Short-term effect of cultivation and crop rotation systems on soil quality indicators in a coastal newly reclaimed farming area. J. Soils Sediments 13, 1335–1350, 2013 .

4-Hazelton, P., Murphy, B., Interpreting soil test results: what do all the numbers mean? CSIRO publishing, Collingwood Victoria, Australia 2016.
